# Supplementary figures and images for: APOE4 accelerates menopause-associated brain metabolic shift and disrupts bioenergetic adaptation
Source: Front Aging Neurosci. 2026 Jun 2;18:1796680. doi: 10.3389/fnagi.2026.1796680 (PMC13269260; doi:10.3389/fnagi.2026.1796680)

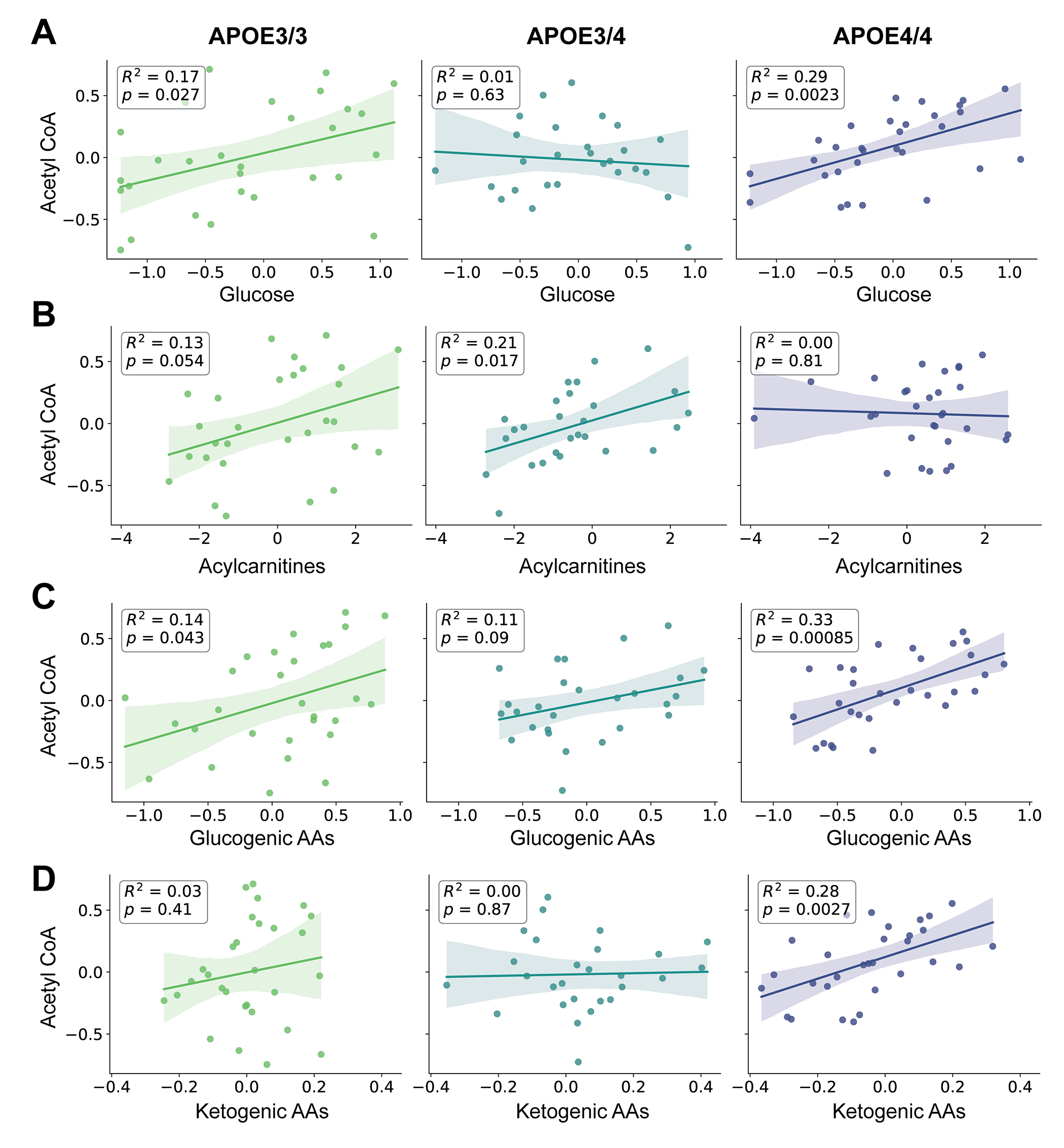

Supplement: Supplementary Figure 1 — Correlations between acetyl-CoA and key metabolic substrates across groups in each APOE genotype: (A) glucose, (B) acylcarnitines, (C) glucogenic amino acids, and (D) ketogenic amino acids. [file Image_1.tif]
